# Fundus Autofluorescence in Inherited Retinal Disease: A Review

**DOI:** 10.3390/cells14141092

**Published:** 2025-07-16

**Authors:** Jin Kyun Oh, Omar Moussa, Byron L. Lam, Jesse D. Sengillo

**Affiliations:** 1 Department of Ophthalmology, Vagelos College of Physicians and Surgeons, Columbia University Irving Medical Center, New York, NY 10032, USA; lukejk.oh@gmail.com (J.K.O.); om2333@cumc.columbia.edu (O.M.); 2 Department of Ophthalmology, Bascom Palmer Eye Institute, School of Medicine, University of Miami Miller, Miami, FL 33136, USA; blam@med.miami.edu

**Keywords:** fundus autofluorescence, inherited retinal diseases

## Abstract

Fundus autofluorescence (FAF) is a non-invasive retinal imaging technique that helps visualize naturally occurring fluorophores, such as lipofuscin, and provides valuable insight into retinal diseases—particularly inherited retinal diseases (IRDs). FAF is especially useful in detecting subclinical or early-stage IRDs and in monitoring disease progression over time. In Stargardt disease, areas of decreased autofluorescence correlate with disease progression and have been proposed as a biomarker for future clinical trials. FAF can also help differentiate Stargardt disease from other macular dystrophies. In retinitis pigmentosa, hyperautofluorescent rings are a common feature on FAF and serve as an important marker for disease monitoring, especially as changes align with those seen on other imaging modalities. FAF is valuable in tracking progression of choroideremia and may help identify disease carrier status. FAF has also improved the characterization of mitochondrial retinopathies such as maternally inherited diabetes and deafness. As a rapid and widely accessible imaging modality, FAF plays a critical role in both diagnosis and longitudinal care of patients with IRDs.

## 1. Introduction

Fundus autofluorescence (FAF) is a non-invasive imaging modality that allows for in vivo assessment of metabolic activity by detecting the distribution of lipofuscin, a fluorophore within the retinal pigment epithelium (RPE) [1,2,3,4,5]. Lipofuscin is produced as a byproduct of the visual cycle as photoreceptor outer segments are shed and subsequently phagocytosed by RPE cells [3,4,5]. Lipofuscin can then be excited using a scanning laser ophthalmoscope to capture an image of the fundus that demonstrates relative areas of lipofuscin density, serving as a reflection of metabolic activity. Given its wide availability and ease of acquisition, FAF serves as a practical and clinically relevant diagnostic tool.

FAF has demonstrated utility across a broad spectrum of retinal disorders, including age-related macular degeneration, central serous chorioretinopathy, chorioretinal inflammatory diseases, vitamin A deficiency, and various toxic or metabolic retinopathies [6,7,8,9,10,11,12]. One of its greatest applications lies in the diagnosis and follow-up of inherited retinal diseases (IRDs). IRDs represent a heterogeneous group of retinal disease that leads to progressive and irreversible degeneration of the photoreceptors and RPE. To date, nearly 300 different genetic etiologies have been identified, many of which have further varied phenotypic presentations [13,14]. FAF has proven effective in identifying and monitoring IRDs, especially when other clinical signs are subtle or absent. As such, there is growing interest in FAF as an outcome measure in IRD clinical trials.

In this review, we explore the clinical applications of FAF in diagnosing and evaluating IRDs, its emerging role in clinical trials, and future directions for its use.

## 2. Physiologic Basis and Clinical Relevance

**Figure 1:**
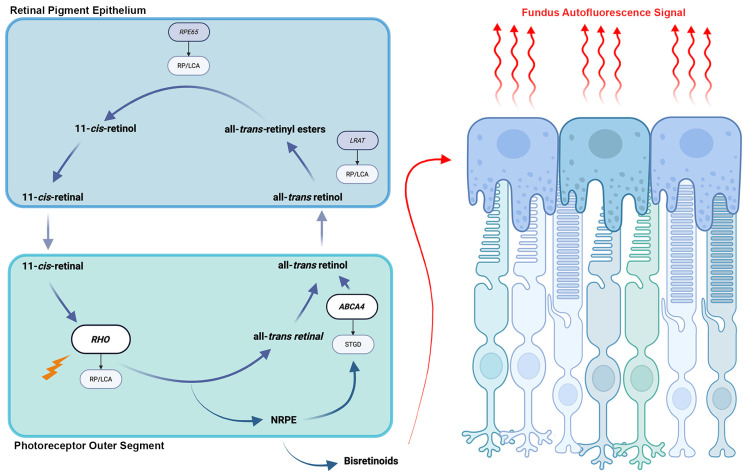
Retinoid visual cycle and the origin of fundus autofluorescence signal. An illustration of the retinoid visual cycle that takes place between the retinal pigment epithelium and the outer segments of photoreceptors is shown. Interruption of the visual cycle at any point can lead to the development of inherited retinal degenerations. Accumulation of bisretinoids in retinal pigment epithelium cells as a byproduct of the cycle is the source of fundus autofluorescence signal.

Lipofuscin accumulates over time in the lysosomal storage bodies of RPE cells and serves as the primary fluorophore in FAF [4,5]. It is produced as part of the visual cycle in the outer segments of photoreceptors and is composed of a mixture of bisretinoids, including A2E, all-trans-retinal dimer, and related isomers [15,16]. The emission spectra of these constituents, which peak at roughly 600nm, form the primary basis for FAF [17,18]. Pathologic deterioration of the visual cycle in photoreceptors, and therefore diminished bisretinoid production or excess accumulation of bisretinoids in the RPE, leads to the ability to detect disease on FAF (Figure 1) [17,18]. In recent years, studies of patients with age-related macular degeneration who exhibit preserved FAF despite outer retinal atrophy have implicated additional fluorophores such as melanin, flavin proteins, collagen, and elastin as potential contributors to the FAF signal [19,20].

Modern FAF utilizes a confocal scanning ophthalmoscope, typically using an excitation wavelength of 488nm, to sweep across the retina [17,18]. The laser is projected through a pinhole and an emission filter with bandwidth of 500–700 nm is utilized to detect emission of fluorescence from excited fluorophores while a barrier filter with cut offs between 500 and 520 nm are used to block reflected light [2]. These images can be obtained within seconds and utilize incredibly low energies for excitation, thereby posing minimal harm. While traditionally obtained in a 30° × 30° or 55° × 55° field, contemporary wide-field scanning laser ophthalmoscopes can even image up to 200° in a single frame, with montaging capabilities extending the field to approximately 220° [Optos California, Optos, Dunfermline, UK) [21,22]. Image processing by the scanning laser ophthalmoscope reduces noise and enhances contrast in order to improve diagnostic utility [20].

Other advancements in autofluorescence imaging include the use of near-infrared autofluorescence (NIR-AF) and quantitative autofluorescence (qAF), both of which offer additional information outside of that provided by conventional FAF. NIR-AF operates on the same principles as FAF but uses an excitation wavelength of 795 nm to sweep the retina, which stimulates a different set of fluorophores, namely melanin [23,24]. Current evidence suggests that RPE melanin in addition to some choroidal fluorophores may be the primary source of NIR-AF singal [23,24]. Like FAF, NIR-AF has shown utility in the diagnosis and monitoring of retinal disease, including IRDs, although it is less widely used in clinical practice [25].

qAF is another application of FAF technology that allows for the measurement and quantification of autofluorescence intensity [26,27]. By incorporating an internal fluorescent reference, qAF corrects for variations in detector sensitivity and transmission and produces numerical values to quantify autofluorescence within a given image [26,27]. This modality allows for longitudinal comparison of images from individual patients, as well as comparison of qAF values between patients with a variety of disease pathology. Although not widely available, qAF has also demonstrated significant utility in the diagnosis and monitoring of IRDs [27].

## 3. Fundus Autofluorescence Features Across Inherited Retinal Disease

Stargardt Disease and Other Macular Dystrophies—Pattern Macular Dystrophy and Best Disease.

Stargardt disease is an autosomal recessive retinal dystrophy caused by biallelic variants in *ABCA4*, which encodes an ATP-binding cassette transporter that plays a crucial role in the visual cycle [28]. Dysfunction of the transporter leads to the accumulation of A2E in photoreceptor outer segments, which are then phagocytosed by RPE cells (Figure 1). Excessive accumulation of lipofuscin in the RPE then exerts toxic effects due to photooxidation and leads to RPE cell death [29]. Characterized by early onset and progressive central vision loss, Stargardt disease is one of the most common IRDs and one of the best studied in its relation to FAF.

**Figure 2:**
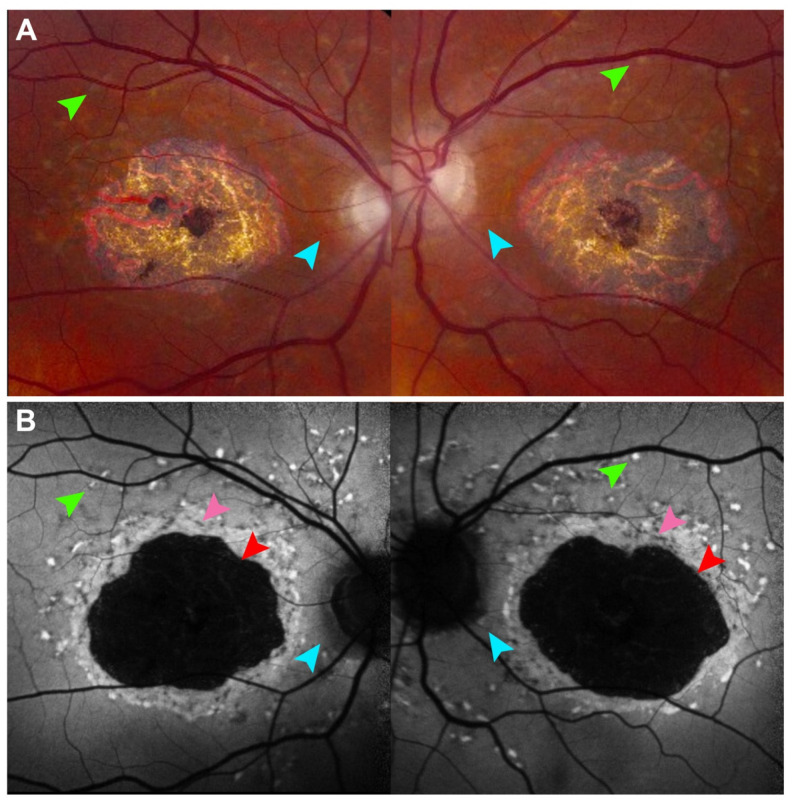
Fundus autofluorescence in Stargardt disease detects features hard to see on color fundus photography. (**A**) 30° field color fundus photography of a 77-year-old patient with Stargardt disease demonstrates central chorioretinal atrophy and faint gray flecks (green arrows) around central atrophy and along the superior arcades. (**B**) 30° field fundus autofluorescence better demonstrates hyperautofluorescent flecks and peripapillary sparing around the optic nerve which is nearly imperceptible on fundus photography (cyan arrows). The well-demarcated definitely decreased autofluorescence (DDAF) (red arrow) has been used in multiple clinical trials to assess treatment efficacy in Stargardt disease. The questionably decreased autofluorescence (QDAF) (pink arrow) refers to areas with intermediate autofluorescence signal loss and represents a transitional stage between healthy retina and advanced atrophy. QDAF regions are often poorly demarcated and progress to DDAF over time.

On FAF, Stargardt disease presents early with hyperautofluorescent deposits, frequently described as pisciform flecks, which correlate to areas of increased lipofuscin accumulation [30]. Key features of disease such as the presence of hyperautofluorescent flecks and peripapillary sparing are frequently better visualized on FAF and may predate the ability to detect disease features on fundus examination or on color fundus photography (Figure 2) [31]. As accumulation of toxic byproducts continue and RPE cells begin to die, these flecks then become atrophic and subsequently hypoautofluorescent. Due to the close relationship between disease pathophysiology and lipofuscin accumulation, FAF has received considerable attention as both a diagnostic tool and biomarker of disease progression.

The Progstar Study Group is a consortium dedicated to characterizing the natural history of Stargardt disease which has highlighted the utility of FAF as a tool to identify disease progression by measuring areas of hypoautofluorescence [32,33,34]. They found that areas of hypoautofluorescence grew, with rates of growth correlating with baseline lesion size, whereby larger lesions grew more rapidly [30,31,32]. These findings were corroborated by both prospective and retrospective studies and suggest that areas of atrophy may serve as both prognostic markers and outcome measurements in clinical trials [32,33,34].

A notable variant in Stargardt disease is the bulls eye maculopathy phenotype, associated with the hypomorphic c.5882G>A, p.(G1961E) allele [35]. This variation appears phenotypically similar to other macular and cone or cone–rod dystrophies. Disease is characterized by the appearance of a hyperautofluorescent bulls eye surrounding central macular atrophy and is known to progress relatively slowly compared to other forms of Stargardt disease. This phenotype presents a diagnostic challenge due to its numerous phenocopies such as *RDS/PRPH2*, *CRX*, or *PROM1* associated macular dystrophy [36]. qAF has proven useful in distinguishing Stargardt disease from these masquerades, as qAF levels are significantly elevated in patients with Stargardt disease compared to both age-matched controls and individuals with other macular dystrophies [37].

*RDS/PRPH2*-associated macular dystrophy is another common IRD, accounting for approximately 5% of all IRDs. *RDS/PRPH2* encodes a photoreceptor specific tetraspanin that plays an important role in outer segment function [38]. Although the precise mechanism remains unclear, these mutations also result in lipofuscin accumulation in the RPE. Variants in *PRPH2* lead to phenotypically variable disease including pattern macular dystrophy, central areolar choroidal dystrophy, and adult vitelliform macular dystrophy [39]. FAF has been suggested to be the most valuable imaging modality in distinguishing the various phenotypic presentations of disease where other imaging modalities such as optical coherence tomography (OCT) may be less sensitive [39]. Notably, qAF levels in *PRPH2*-associated disease are moderately elevated compared to that of other macular dystrophies but remain below those observed in Stargardt disease [40]. The underlying mechanism of this intermediate accumulation is poorly understood [40].

Best Disease, or Best vitelliform macular dystrophy, is the most common autosomal dominant macular dystrophy and is characterized by mutations in *BEST1,* which encodes a calcium gated ion channel in the RPE [41]. Loss of BEST1 function has also been associated with accumulation of lipofuscin in the RPE, similar to Stargardt disease, although the mechanism remains poorly understood [41]. Best disease is characterized by the appearance of a vitelliform lesion in the macula that expands before involuting into an atrophic scar. Despite the central location of disease pathology, visual acuity tends to remain relatively well preserved until the later stages of the disease [42]. On FAF, this lesion initially appears hyperautofluorescent and becomes progressively hypoautofluorescent as the lesion becomes atrophic [43,44]. Areas of subretinal fluid may appear as a dependent hyperautofluorescent level within the vitelliform lesion [43,44]. Lesion area and diameter have been shown correlate with disease progression and may represent potential outcome measurements for future clinical trials [43,44,45].

## 4. Retinitis Pigmentosa and Other Rod–Cone Dystrophies

**Figure 3:**
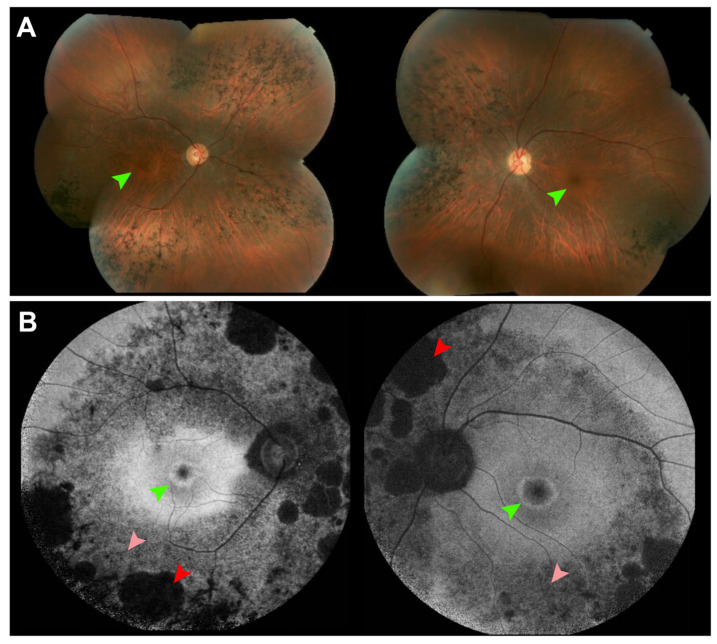
Hyperautofluorescent rings in retinitis pigmentosa seen on fundus autofluorescence compared to color fundus photography. (**A**) Montage color fundus photographs of a 36-year-old patient with autosomal dominant retinitis pigmentosa due to mutations in *RHO* demonstrate waxy pallor of the optic nerve, vascular attenuation and nasal and inferior bone spicules. (**B**) 55° field fundus autofluorescence demonstrates a central hyperautofluorescent ring, smaller in the right eye than the left, which is not seen on color fundus photographs (green arrows). Areas of both definitely decreased autofluorescence (red arrows) and questionably decreased autofluorescence can be seen (pink arrows).

Retinitis pigmentosa (RP) encompasses a heterogeneous and diverse group of IRDs characterized by degeneration of rod followed by cone photoreceptors. Over 80 different genes have been implicated in disease pathogenesis, posing a significant challenge in diagnosis, monitoring of disease, and identification of biomarkers for clinical trial outcomes [46]. RP is typically characterized by a triad of bone spicule-like pigment migration, waxy pallor of the optic disk, and vascular attenuation on clinical examination. On FAF, RP typically presents with a central ellipsoid hyperautofluorescent ring, which has been shown to progressively shrink with disease progression, and diffuse hyperautofluorescence in the periphery (Figure 3). This ring, which is seen in over 50% of patients with RP and is undetectable on fundus examination, has been frequently studied as a disease marker for progression especially as it correlates well with other structural and functional modalities [47,48,49].

Changes in the hyperautofluorescent ring correlate strongly with retinal function, as the hyperautofluorescence demarcates the boundary between diseased and healthier photoreceptors [50,51,52]. As disease progresses, the ring begins to shrink and corresponds to evolving central vision loss [50,51,52]. Given the genotypic and phenotypic heterogeneity of disease, many efforts have been made to classify this change based on subgroupings such as inheritance pattern or by affected gene [51]. Prior studies have demonstrated that X-linked RP progresses faster than autosomal recessive RP, which in turn progresses faster than autosomal dominant RP, as evidenced by a more rapid decrease in ring size on FAF in X-linked RP [51].

Studies by individual gene are often limited to more common genetic etiologies of RP, including disease caused by *RHO* or *USH2A*, due to the limited number of affected patients. Mutations in *RHO*, the most common cause of autosomal dominant RP, lead to a slowly progressive disease, making RP due to mutations in *RHO* a target in gene therapy trials [53]. Prior studies have shown changes in hyperautofluorescent ring area as well as vertical and horizontal diameter to be relatively slow compared to other genetic etiologies of RP [53]. Similarly, mutations in *USH2A*, the most common cause of autosomal recessive RP, is also a focus of therapeutic interest [54]. The Foundation Fighting Blindness Consortium recently conducted a natural history study (RUSH2A) to identify clinically relevant endpoints for clinical trials [55]. Although FAF was not used, ellipsoid zone (EZ) length on OCT was used, which is highly correlated with ring diameter on FAF based on prior study with correlation coefficients upwards of 0.99 [54]. This suggests that although not used by this study, FAF changes likely remain a viable outcome measurement for clinical trials.

Despite the classic hyperautofluorescent ring appearance on FAF, other forms of ring morphology have also been described including double rings and irregular or asymmetric rings [56,57,58]. Double hyperautofluorescent rings have been described in disease associated with variants in *RHO*, *RPGR*, *USH2A*, as well as *NR2E3*, while irregular rings are more common with variants in *RHO*, *RP1*, and *EYS* [56,57,58]. These irregular rings may limit the applicability of hyperautofluorescent rings on FAF as potential outcome measures given their irregular morphology and difficulty characterizing progression; however, they may serve as diagnostic aids in narrowing genetic diagnoses.

The absence of autofluorescence on FAF can also be a critical diagnostic feature of RP caused by mutations in the visual cycle. The production of lipofuscin is dependent on the activity of the visual cycle within photoreceptors; therefore, in disease caused by variants in the visual cycle such as *RPE65* or *LRAT*, severely diminished FAF has been described [59,60]. A recent study confirmed that mutations across all genes in the visual cycle may be associated with diminished FAF, which was corroborated by the use of qAF [59]. Notably, the advent of gene therapy for *RPE65*-mediated Lebers congenital amaurosis provided rare insight into the changes in FAF following treatment. A case report demonstrated restored qAF in a 9-year-old girl with biallelic mutations in *RPE65* following treatment with voretigene neparvovec, supporting the role of FAF not only as a diagnostic aid but also as a potential biomarker of treatment [61]. More recently, longer-term follow-up studies have shown that nearly half of treated patients develop progressive chorioretinal atrophy, manifesting as circular areas of hypoautofluorescence that correspond to areas of subretinal bleb formation [62,63].

FAF in RP is particularly versatile when used alongside other structural and functional modalities. In addition to OCT, both OCT angiography and adaptive optics have provided valuable insights into disease pathogenesis [48,49]. The boundaries of hyperautofluorescent rings in RP have been shown to correlate with the blurring of cone photoreceptors on adaptive optics, reflecting a marked reduction in macular cone density [48]. Similarly, areas of preserved hyperautofluorescence are associated with intact retinal capillary density, whereas regions of decreased autofluorescence demonstrate reduced capillary density [49]. These findings underscore the value of FAF as a complementary tool given its strong correspondence with multiple ophthalmic imaging and diagnostic modalities.

## 5. Choroideremia

**Figure 4:**
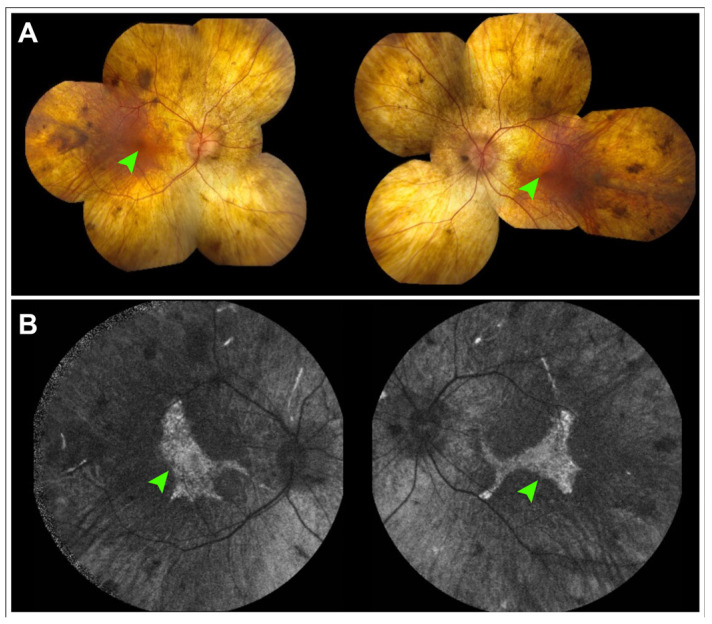
Color fundus photographs and fundus autofluorescence in choroideremia. (**A**) Montage color fundus photographs of a 49-year-old patient with choroideremia demonstrates diffuse chorioretinal atrophy. (**B**) 55° field fundus autofluorescence demonstrates a small island of centrally preserved relative hyperautofluorescence that correlates to preserved photoreceptors (green arrows). The area of this central island has been measured as a biomarker of disease progression and is challenging to measure on fundus photography.

Choroideremia is an X-linked recessive IRD characterized by nyctalopia and progressive peripheral visual field loss [64]. Caused by mutations in *CHM*, which codes for REP-1, the disease leads to progressive degeneration of the RPE followed by secondary loss of the photoreceptors and choroid [64]. On examination, choroideremia presents as scalloped areas of chorioretinal atrophy across the posterior pole but often sparing the foveal center. FAF typically reveals areas of granular hyper- and hypoautofluorescence surrounded by broad patches of hypoautofluorescence corresponding to areas of atrophy (Figure 4).

FAF has been of particular interest in the evaluation of choroideremia, with several studies characterizing rate of autofluorescence loss and comparing it to other structural and functional outcome measurements such as outer nuclear layer thickness, EZ length, and sensitivity on microperimetry [65,66,67]. A recent natural history study by the NIGHT study group found that the area of involvement on FAF was a more sensitive measure of disease progression than best-corrected visual acuity, suggesting that disease area on FAF may be a viable outcome measurement for clinical trials [68]. Studies by the NIGHT group have also shown that disease progression in choroideremia may have directional preference, more prominently affecting the nasal subfields than others [65].

FAF also plays a role in the diagnosis and monitoring of heterozygous carriers of *CHM*. Although often asymptomatic, carriers may develop nyctalopia, and in severe cases, exhibit features and symptoms similar to actual disease [69,70]. Carriers with intermediate or severe phenotypes have disease that more closely resembles true choroideremia and FAF can be used to monitor disease progression. In carriers with milder phenotypes, patches of hyperautofluorescence alternating with hypoautofluorescence are seen on FAF, often described as a “mud splatter” pattern [69]. In this context, FAF can aid in diagnosing carrier status even in the absence of genetic testing and may inform family planning decisions.

## 6. Mitochondrial Disease

Mitochondrial disease has long been associated with phenotypically variable retinopathy. One of the most well-characterized mitochondrial retinopathies is maternally inherited diabetes and deafness (MIDD), which is frequently caused by a common point mutation in *MT-TL1*, m.3243A>G. MIDD typically presents as a pattern-like dystrophy with RPE and photoreceptor atrophy resembling the geographic atrophy seen in AMD [71]. On FAF, disease is characterized by hyperautofluorescent flecks, resembling those seen in Stargardt disease, radiating from central areas of hypoautofluorescence corresponding to chorioretinal atrophy. FAF in MIDD has proved valuable in both diagnosis and monitoring disease progression, especially when compared to examination or fundus photography alone [72]. One study by Ovens et al. demonstrated that areas of RPE atrophy can be readily tracked on FAF and may serve as relevant biomarkers in clinical trials [73]. Others have also demonstrated that patients with multifocal atrophy have more rapid disease progression, suggesting that baseline disease features may have prognostic value [74].

FAF is also valuable in the diagnosis and monitoring of other types of mitochondrial retinopathies. Birtel et al. proposed a classification system dividing mitochondrial retinopathies into three groups [75]. Type 1 is described as the small foci of hyper- and hypoautofluorescence on FAF that are frequently associated with large mitochondrial deletions, classically seen in Kearns Sayre and the chronic progressive external ophthalmoplegia spectrum of disease [75,76]. Type 2 is the phenotype most commonly associated with MIDD with hyperautofluorescent flecks and central areas of hypoautofluorescence [75]. Type 3 is associated with a diffuse granular hypoautofluorescence that extends outside of the arcades. This phenotype has been associated with variants in several genes, including *MT-TL1*, *MT-RNR1*, and *MT-ATP6* [75]. In recent years, mutations in *RNT4IP1*, *SSBP1*, and *WFS1*—genes that play crucial roles in mitochondrial function—have been associated with both mitochondrial retinopathy and optic neuropathy [77,78,79,80].

Mitochondrial retinopathies are of particular interest due to the insight they may provide into more common ophthalmic conditions such as age-related macular degeneration [81,82]. Several studies have suggested that accumulation of mitochondrial damage in RPE cells is a key feature of disease pathogenesis [81,82,83]. Similarities in features on FAF have also been noted. Hyperautofluorescence at the borders of chorioretinal atrophy is seen in both AMD and mitochondrial retinopathies such as MIDD and heralds impending expansion of atrophy [73]. Consequently, keen interest has been paid to mitochondrial retinopathies to provide insights into improving our understanding of AMD.

## 7. Fundus Autofluorescence as a Quantitative Outcome Measure in Inherited Retinal Disease Natural History Studies and Clinical Trials

Natural history studies are critical precursors to therapeutic clinical trials for IRDs as they establish how disease progresses in the absence of treatment. Recently, such studies have become increasingly common, particularly for genetically defined forms of RP, with many incorporating FAF to monitor disease progression. In the past two years alone, multiple natural history studies for various forms of RP have been described [84,85,86,87].

FAF is an established primary outcome measure in clinical trials for IRDs, particularly for Stargardt disease. FAF enables objective quantitative assessment of retinal atrophy to evaluate whether treatment slows disease progression compared to untreated eyes.

Well-demarcated contiguous definitely decreased autofluorescence (DDAF) has been used in multiple clinical trials to assess treatment efficacy in Stargardt disease (Figure 2B). DDAF refers to areas of essentially complete FAF signal loss (≥90% darkness relative to the optic nerve head) and corresponds anatomically to geographic atrophy on OCT characterized by loss of RPE cells and overlying photoreceptors. In some Stargardt patients, the area of EZ loss exceeds the area of DDAF, suggesting that photoreceptor degeneration may precede retinal pigment epithelium atrophy [88].

A significant reduction in the progression of size of well-demarted DDAF with treatment is considered evidence of therapeutic benefit. However, contiguous well-demarcated DDAF is observed in only a subset of Stargardt patients and signifies a moderately advanced stage of disease.

Questionably decreased autofluorescence (QDAF) refers to areas with intermediate autofluorescence signal loss (50–90% darkness relative to the optic nerve head) and represents a transitional stage between healthy retina and advanced atrophy. QDAF regions are often poorly demarcated and progress to DDAF over time. Both QDAF area and the total autofluorescence area of DDAF and QDAF are being explored as potential outcome measures in clinical trials for Stargardt disease. However, the poorly defined borders of QDAF lesions pose challenges to obtaining reliable and reproducible measurements [89,90]. This becomes increasingly important as automated segmentation and artificial intelligence models are being proposed as adjuncts to human graders in clinical trials [89,90].

## 8. Conclusions

FAF is an indispensable tool in the diagnosis and monitoring of numerous retinal diseases. Across the spectrum of IRDs, including Stargardt disease and pattern dystrophies, RP, choroideremia, and mitochondrial retinopathies, FAF has demonstrated consistent utility in disease diagnosis, monitoring of progression, and guiding prognostication. Several biomarkers for potential clinical trials, such as changes in hyperautofluorescent ring size or area of hypoautofluorescent atrophy, are increasingly being considered as potential outcome measurements in clinical trials. As our understanding of IRDs deepens, FAF will likely remain a mainstay of both clinical care and research.

## Data Availability

Not applicable.
